# An Efficient Strategy for Enhancing the Adsorption of Antibiotics and Drugs from Aqueous Solutions Using an Effective Limestone-Activated Carbon–Alginate Nanocomposite

**DOI:** 10.3390/molecules26175180

**Published:** 2021-08-26

**Authors:** Ahmed H. Ragab, Hala S. Hussein, Inas A. Ahmed, Khamael M. Abualnaja, Najla AlMasoud

**Affiliations:** 1 Department of Chemistry, Faculty of Science, King Khalid University, Abha 62224, Saudi Arabia; ahrejab@kku.edu.sa; 2 Chemical Engineering & Pilot Plant Department, Engineering Division, National Research Center, Cairo 11865, Egypt; hala.hussein21@yahoo.com; 3 Department of Chemistry, College of Science, Taif University, Taif 21944, Saudi Arabia; k.ala@tu.edu.sa; 4 Department of Chemistry, College of Science, Princess Nourah Bint Abdulrahman University, Riyadh 11671, Saudi Arabia; nsalmaoud@pnu.edu.sa

**Keywords:** amoxicillin (AMX), diclofenac (DCF), limestone, activated carbon, alginate, adsorption

## Abstract

Based on the adsorption performance of a porous nanocomposite with limestone (LS), activated carbon (AC) and sodium alginate (SG), a unique, multifunctional LS–AC–SG nanocomposite absorbent was designed and prepared for extracting antibiotics and drugs from aqueous solutions. The composite exhibited the following advantages: quick and simple to prepare, multifunctionality and high efficiency. Amoxicillin (AMX) and diclofenac (DCF) were chosen as the conventional antibiotic and the drug, respectively. The prepared nanocomposite’s physicochemical characteristics were calculated through numerous characterization methods. The structure of the surface was made up of interconnected pores that can easily confine pollutants. The surface area was measured to be 27.85 m^2^/g through BET analysis. The results show that the maximum absorption capacity of amoxicillin and diclofenac was 99.6% and 98.4%, respectively, at a contact time of 40 min. The maximum removal of amoxicillin and diclofenac was reached at pH = 2. Adsorption analysis revealed that adsorption isotherm and kinetic data matched the pseudo-first-order kinetic and the Langmuir isotherm models. The results imply that the synthesized nanocomposites have the capacity to remove amoxicillin (AMX) and diclofenac (DCF) from aqueous solutions.

## 1. Introduction

Antibiotics and drugs are chemical compounds with a wide spectrum of applications in humans and veterinary medicine. They are used for treatment of diseases caused by various bacterial infections in addition to their wide usage in animal farming and aquaculture activities for disease prevention and growth promotion purposes [1,2]. The excessive use of these pharmaceutical materials increases the amount of antibiotic and drug residues released into the environment. As a result, the contamination of the environment caused by antibiotics and drugs has recently received more attention. Likewise, the pollution caused by the widespread release of these substances into the aquatic environment, the toxic and long-term effects of those pharmaceuticals persist, and the development of advanced and sensitive analytical tools is ongoing [3]. The surge in antibiotic concentration levels in the aquatic environment is caused by the incomplete metabolization in the human and animal body, as antibiotics are generally excreted with urine and animal manure. Consequently, they are expelled as water soluble conjugate compounds or metabolites [4,5]. Additional sources of pollution are the agricultural runoff and the disposal of unused antibiotic drugs obtained from manufacturing industries [6]. The research has shown antibiotics and drugs to be effective and present in effluents of wastewater treatment plants such as hospital wastewater, surface and ground water, industrial effluents, drinking water and sediments [7,8,9,10].

Amoxicillin (AMX) is one of the most widely employed commercial penicillins due to its high bacterial resistance and large spectrum against a wide range of microorganisms [11,12]. It can be found in wastewater coming from pharmaceutical plants and hospitals, which causes skin disorders and microbial resistance among pathogen organisms. It has been reported that about 30−90% of AMX are discharged into the environment through human and animal excrements [13] and the presence of AMX in the ng L^−1^ to mg L^−1^ concentration ranges in surface water, domestic and industrial wastewater. Since resistant bacteria can cause diseases that are not treatable with traditional antibiotics, amoxicillin waste must be treated before its disposal [14]. One of the most frequently used nonsteroidal anti-inflammatory drugs (NSAID) in the world is diclofenac (DCF) [15]. It is well-known as a sodium or potassium salt in water systems, such as surface and drinking water, wastewater from groundwater, and treatment plant effluents [16]. Actually, large-scale consumption of DCF enhanced its existence in wastewater, which badly impacts living organisms [17]. According to the reported data, the measured values of diclofenac in municipal wastewater can be up to 7.1 μg·L^−1^ [18]. Thus, the removal of DCF from water systems in imperative.

There exist several approaches to removing pharmaceutical compounds from the environment, including absorption [19,20], chemical oxidation [21] and microbial degradation [22], etc. Thanks to its high efficiency [23] and antitoxic nature [24], adsorption is an excellent method for treating wastewater with low concentrations of antibiotics.

Thanks to its high adsorption capacity, surface area and microporous structure, activated carbon is widely used as an adsorbent for discharging pollutants from aqueous or gaseous phases [25,26]. However, activated carbon is expensive and has a number of issues with regeneration [27]. Therefore, to resolve the issue, low-cost materials can be used, for example, rice husk, peat, pine wood and coconut shells.

Limestone is a popular adsorbent due to its low cost and wide availability in nature. It has been demonstrated that limestone can remove heavy metals such as iron via filtration [25,28]. Additionally, its heterogeneous surface, buffering quality, secondary binding site and repurposing properties are very useful [28]. Thus, limestone can be employed as a low-cost adsorbent in water treatment.

Brown seaweeds feature a natural polysaccharide called alginate [25,29]. It is an efficient, inexpensive and non-toxic material generally used as one of the biopolymers for pollutants release from aqueous solutions. This polymer is very useful in textile and paper industries for developing on the surface of paper and clothing. It has been found that when treating turbid water with calcium alginate as a coagulant, 98% of the turbidity can be removed [30]. Alginate is further used as a binder for composite media and for the flocculation process in water treatment.

Limestone, alginate and activated carbon are not always successful in water treatment on their own; the combination of the three create an effective composite material for removing antibiotic and drug residues from aqueous solutions. In this study, their combined roles within the LS–AC–AlG nanocomposite was expected to show a synergistic effect on the efficient removal of AMX and DCF, which is closely related to the enhancement of adsorption from water matrices. The optimum conditions such as dosage, sample pH concentration were determined using the response surface methodology. Langmuir and Freundlich isotherm models, in addition to kinetic models, were used to analyze the experimental equilibrium data.

## 2. Results and Discussion

### 2.1. LS–AC–SG Structure and Characteristics

#### 2.1.1. FTIR Study

**Figure 1:**
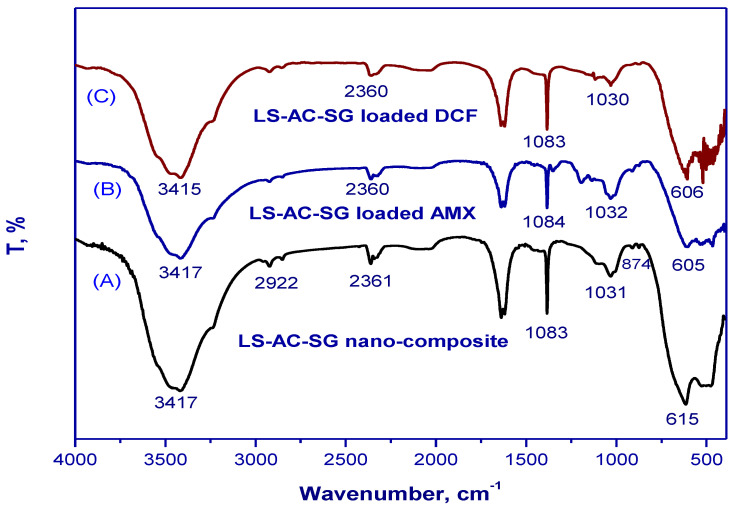
FTIR spectra of (**A**) the LS–AC–SG nanocomposite, (**B**) LS–AC–SG-loaded AMX, (**C**) LS–AC–SG-loaded DCF.

Figure 1 illustrates the Fourier-transform infrared spectra of the LS–AC–SG nanocomposites before and after amoxicillin and diclofenac adsorption. A wide band corresponding to the phenolic group appeared at approximately 3416 cm^−1^ and correlated with O–H (hydroxyl) groups. The peak at 2361 cm^−1^ corresponded to acid O–H [31]. Moreover, a peak that appeared at 615 cm^−1^ was related to C–H out-of-plane bending in benzene derivatives [32]. After the adsorption, numerous functional groups adjusted to different bands or disappeared. The band at 3417, 2361, 1031 and 615 cm^−1^ shifted to 3415, 2360, 1030 and 606 cm^−1^, respectively, after adsorption of diclofenac (DCF). The band at 2361, 1083, 1031 and 615 cm^−1^ shifted to 2360, 1084, 1032 and 605 cm^−1^, respectively, after adsorption of amoxicillin (AMX). The peak at 2922 cm^−1^, on the other hand, vanished after adsorption.

#### 2.1.2. SEM Study

**Figure 2:**
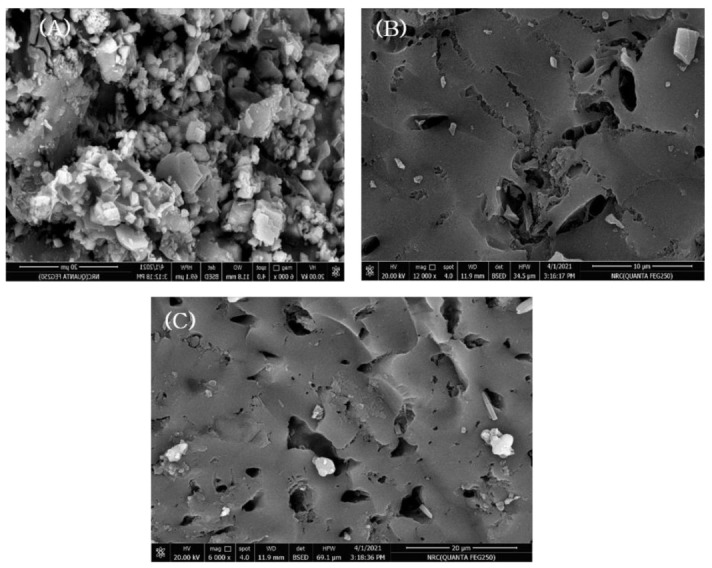
SEM of (**A**) LS–AC–SG nanocomposite, (**B**) LS–AC–SG-loaded AMX, (**C**) LS–AC–SG-loaded DCF.

The SEM analysis of the synthesized LS–AC–SG nanocomposites is presented in Figure 2 before and after amoxicillin and diclofenac adsorption. More pores were observed in the surface morphologies in Figure 2A than in Figure 2B,C, suggesting that the nano-composite has sufficient room for the adsorption process to happen. Based on Figure 1B,C, due to the AMX and DCF that covered the nanocomposite, fewer pores appeared. Earlier studies on the activated carbon–limestone–alginate composite also described the same results [33,34].

#### 2.1.3. Transmission Electron Microscopy Study

**Figure 3:**
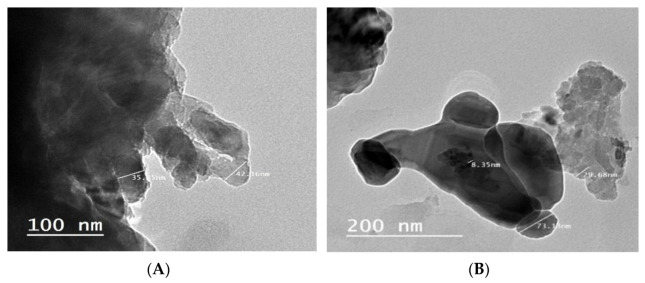
TEM analysis of the LS–AC–SG nanocomposite: (**A**) 100 nm, (**B**) 200 nm.

The TEM images of the LS–AC–SG nanocomposite are provided in Figure 3. The TEM micrographs show that the constructed LS–AC–SG nanocomposite exhibited a multilayer structure with a crystal size of ≈20 nm, as shown in Figure 3. This irregular surface suggests that nucleation took place, in other words, the film covering the particles nucleated on the alginate [35].

#### 2.1.4. Adsorption–Desorption Measurements

**Figure 4:**
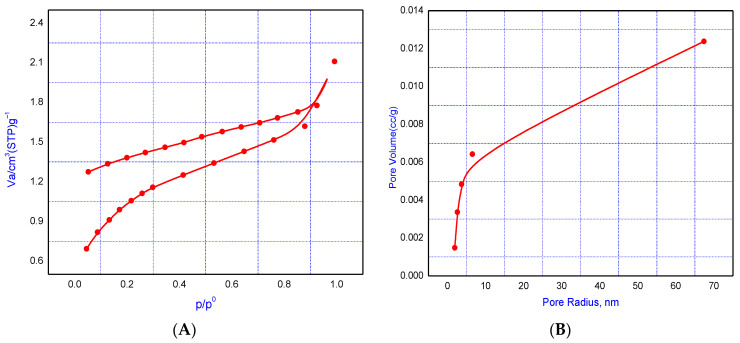
(**A**) Adsorption–desorption isotherm, (**B**) pore size distribution of the LS–AC–SG nanocomposite.

BET adsorption–desorption measurements were used to investigate the characteristics of the LS–AC–SG nanocomposite. The composite’s constituents’ N_2_ adsorption–desorption isotherms and pore size distribution curves at 77 K are shown in Figure 4A,B. The surface area data showed that the pore volume and surface area of the nanocomposite (LS–AC–SG nanocomposite) were 2.469 cm^3^ g^−1^ and 27.859 m^2^ g^−1^, respectively, as listed in Table 1.

### 2.2. Performance of the LS–AC–SG Nanocomposite

#### 2.2.1. Effect of pH

An extremely significant factor that affects the removal efficiency of an adsorbent in wastewater treatment is the pH of the solution since the adsorption efficiency is influenced by the pH of the medium. The impact of particular adsorbents on the adsorption of AMX and DCF on the pH of the solution can be expressed in terms of the dissociation of AMX, DCF and the surface charge of the adsorbent. The ionization state of AMX and DCF molecules is determined by the pH of the solution phase, which can be positive, negative or neutral depending on the pH values. The interaction mechanisms of LS–AC–SG with AMX and DCF are explained as follows; in a strong acidic environment, most of the surface adsorption sites of adsorbents are protonated and exhibit positive charges. AMX^−^ or DCF^−^ was electrostatically attracted by the deprotonated positive-charge adsorption sites, so the removal percent was high at pH value equal 2. At pH 2, the maximum removal efficiency was found to be 99.6% for AMX and 98.4% for DCF. As the pH increased, surface adsorption sites were deprotonated, and negative charge sites increased, and electrostatic repulsion was also generated between adsorbates and adsorbents; therefore, the increased trend of AMX and DCF percent removal was low. The degree of deprotonation was enhanced at pH higher than 2 and the repulsion between negative charge sites and anions was pretty intense, resulting in a sharp decrease in the percent removal of AMX and DCF at pH 10. Based on the above results, electrostatic attraction played an indispensable role in AMX and DCF adsorption and pH 2 is optimum for the AMX and DCF absorption process.

#### 2.2.2. Contact Time Effect

**Figure 5:**
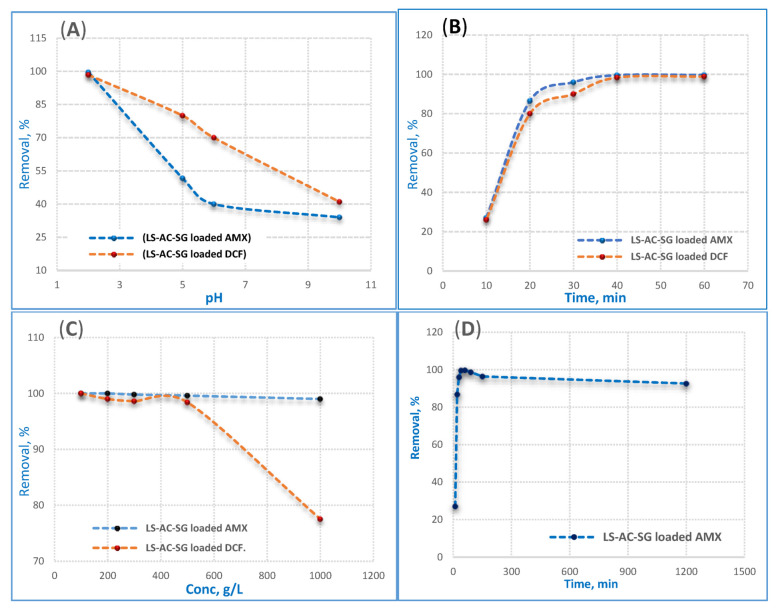
Influences of (**A**) pH, (**B**) contact time and (**C**) initial (AMX and DCF) concentration on the adsorption of AMX and DCF by 0.05 g/25 mL of the nanocomposite at pH 2 and a contact time of 40 min, (**D**) long-term stability of LS–AC–SG in a low pH (2) solution.

The effect of contact time on AMX and DCF adsorption on the LS–AC–SG nanocomposite at concentrations of 500 ppm using 0.05 g/25 mL of the nanocomposite and pH 2 is presented in Figure 5B. Firstly, the AMX and DCF adsorption process was extremely quick within the initial 20 min and then dropped slowly till reaching the equilibrium. The adsorbed amounts increased with the increase in contact time. This sensation may be attributed to the powerful interaction between the LS–AC–SG nanocomposite surface and AMX, DCF molecules for the duration of the first 20 min (electrostatic forces); then, the physisorption of AMX–DCF reached equilibrium via the van der Waals forces [36]. Figure 5B confirms two kinetic regimes: the first region is described by a high rate of adsorption. This is because of the high number of sites originally available on the LS–AC–SG nanocomposite surface (charged adsorption sites), indicating a rapid increase at the start of the adsorption procedure, followed by a slower uptake till the equilibrium state, while in the second part, the adsorption of AMX and DCF onto the LS–AC–SG nanocomposite surface remained constant beyond 40 min, leading to reduced interaction due to the limited number of surface binding sites. This result could be attributed to an increase in electrostatic interactions between the surfaces of adsorbents and adsorbates.

#### 2.2.3. Effect of the AMX and DCF Concentrations

The effect of AMX and DCF concentrations on the adsorption technique was investigated at concentrations ranging from 100 to 1000 ppm using 0.05 g/25 mL of the nanocomposite, pH 2, and a contact time of 40 min, as shown in Figure 5C. The findings show that the increase in the concentration of AMX and DCF has a negative effect on the removal efficiency. That is to say, with augmentation of the initial pollutant concentration, the efficiency of adsorption decreases because as the initial pollutant concentration increases, its remaining value also increases, and consequently its removal efficiency is diminished. Moreover, the saturation of the adsorbent surface at high concentrations of pollutants also decreased the adsorption ability. The outcomes demonstrated that by increasing the AMX and DCF concentrations from 100 mg/L to 1000 mg/L, the adsorption capacity decreased from 99.9% to 99% for AMX and from 99.9% to 77.5% for DCF; that means the composite is perfect for AMX.

#### 2.2.4. Long-Term pH Stability

The long-term stability of LS–AC–SG in a low pH solution, in particular in a pH 2 solution, was evaluated as shown in Figure 5D. Figure 5D indicates that the performance of LS–AC–SG was stable over the full time period. It can be noticed that the performance of LS–AC–SG was nearly unaffected. In the latter time, after 20 h, a small drop in the permeance (~0.9%) was observed. Overall, we can consider the LS–AC–SG nanocomposite to be stable, with a final performance of 92.6%.

### 2.3. Kinetic Models

Three distinct kinetic models were used to investigate the adsorption kinetics of the LS–AC–SG nanocomposite, including the pseudo-first-order, pseudo-second-order and intraparticle diffusion models [37]. The optimal conditions were established as pH 2, LS–AC–SG nanocomposite mass of 0.05 g/25 mL, a contact time of 40 min, and 500 ppm of AMX and DCF as the starting concentration.

#### 2.3.1. Pseudo-First-Order Reaction Kinetics

**Figure 6:**
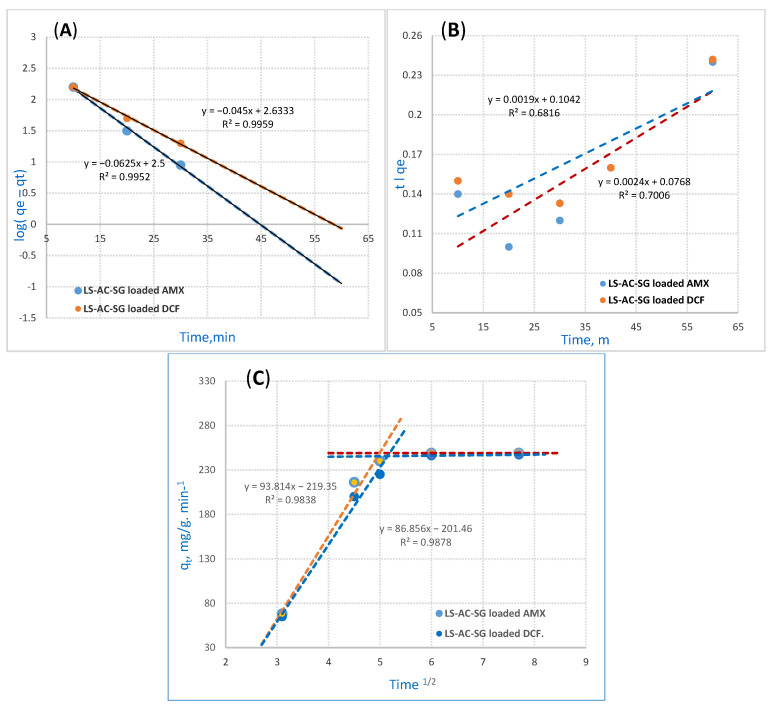
Adsorption kinetics: (**A**) pseudo-first-order reaction (PFORE), (**B**) pseudo-second-order reaction (PSORE), (**C**) Mories–Weber equation for AMX and DCF on the LS–AC–SG nanocomposite (sorption time, 40 min; sorbent dosage, 0.05 g/25 mL, pH = 2).

The PFOR reaction kinetics equation is detailed in Figure 6 [38]. The equation applied for the instant initial phase is as follows:

Log (q_e_ − q_t_) − log q_e_ = −K_ads_ t/2.303 (1)

where q_t_ (mg/g) represents the adsorption capacity at time t while K_ads_ (min^−1^) stands for the rate constant of PFOR adsorption. In this research, a linear relationship was noticed for the adsorption of AMX and DCF ions onto the LS–AC–SG nanocomposite. The values of q_e_ and k_ads_ were measured from the slope and intercept by plotting log (q_e_ − q_t_) versus t. The PFOR kinetics are illustrated in Figure 6A. PFOR correlation coefficients (R^2^) for the LS–AC–SG nanocomposite in Table 2 maintained high values. The outcomes exhibited high correlation coefficients (R^2^ = 0.9952, 0.9959) for AMX and DCF. The statistics imply that the AMX and DCF adsorption suited the pseudo first-order kinetics.

#### 2.3.2. Pseudo-Second-Order Reaction

The PSOR kinetic model [39] is illustrated in the following equation:

t/q = 1/K_2_q_e2_ + t/q_e_ (2)

where K_2_ (g/mg/min) stands for the PSOR rate constant, illustrated in Figure 6B. The slopes and intercepts determine the values of the rate constant K_2_ and equilibrium adsorption capacity q_e_, including correlation coefficient (R^2^) as well when t/q_t_ is plotted versus t. The outcomes exhibit a correlation. Table 3 details these values. The collected data show that the pseudo-second-order model has a poor fit for the adsorption of AMX and DCF onto the LS–AC–SG nanocomposite.

#### 2.3.3. Mories–Weber Kinetic Equation

The intraparticle mass transfer diffusion can be described by the Mories–Weber Equation (3) [40] and is shown in Figure 6C:

q = K_d_ (t)^1/2^ (3)

where q (g/g) stands for the uptake of metal ions, whereas K_d_ represents the intraparticle mass transfer diffusion rate constant and t^1/2^ stands for the square root of time. It would only happen in the shorter stage if the adsorption data and the intraparticle diffusion overlapped. The Morris–Weber equation in Figure 6C shows that the first portion is linear, which is related to the boundary layer effect. However, the second part could be linked to the intraparticle diffusion effect [41]. This signifies that almost all sorption happens in the first 40 min, with a definite linear direction; this confirms that nanocomposites’ porosity surpasses the resistance affecting intraparticle diffusion [42]. The intraparticle diffusion rate constant value K_d_ was gauged to be 93.8 and 86.8 (g/g·min^−1^) for AMX and DCF adsorption onto the LS–AC–SG nanocomposite, suggesting AMX and DCF ions move to the composite. The kinetic modeling with the PFOR, PSOR and Mories–Weber equations are detailed in Table 2.

### 2.4. Isotherm Model

Isotherm studies are essential to interpreting the adsorption process adequately [43]. Among these isotherm models, the Langmuir, Freundlich and Dubinin–Radusekevisch–Kanager models were implemented to examine the adsorption process. The experimental conditions were adjusted to pH 2, a mass of 0.05 g/25 mL of the LS–AC–SG nanocomposite, a contact time of 40 min and an AMX and DCF concentration of 500 ppm.

**Figure 7:**
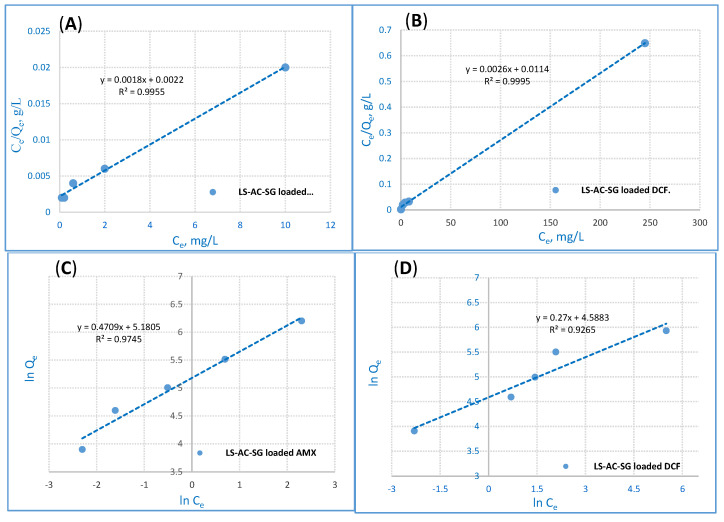
Langmuir adsorption for (**A**) AMX ions removal and (**B**) DCF ions removal and Freundlich adsorption for (**C**) AMX ions removal and (**D**) DCF ions removal from the LS–AC–SG nanocomposite (sorption time: 40 min; sorbent dosage: 0.05 g/25 mL, pH = 2).

The adsorption of any substance on a homogenous surface with insignificant interaction between the adsorbed molecules was elucidated via the Langmuir isotherm [44]. The model postulates a uniform uptake on the highest-adsorption surface according to the monolayer’s saturation level. The Langmuir linear equation model is illustrated as follows [45]:

C_e_/q_e_ = 1/K_L_·q_max_ + (1/q_max_)·C_e_ (4)

where K_L_ (L·mg^−1^) stands for the sorption heat’s adsorption capacity of the monolayer and q_max_ (mg·g^−1^) represents the maximum adsorption capacity. The Langmuir adsorption isotherm established on the monolayer adsorption through the adsorption process is illustrated in Figure 7A,B. The Langmuir model explains the equilibrium uptake of the adsorbents’ homogenous surface.

The Freundlich model is among the first empirical equations compatible with the exponential distribution of active centers, especially for heterogeneous surfaces [46,47], illustrated as follows:

ln q_e_ = ln K_f_ + 1/*n* ln C_e_ (5)

where K_f_ represents the capacity of adsorption, *n* stands for intensity and K_f_ is a vital and relative measure of the capacity of the adsorption; it stands for a favorable adsorption extent. When *n* exceeds 1, adsorption is deemed suitable [48]. The outcomes show that the Langmuir model fitted the experimental data of the LS–AC–SG nanocomposites better than the Freundlich model. The correlation coefficient (R^2^) values are detailed in Table 3. The values of R^2^ of the Langmuir model data for both AMX and DCF adsorption were 0.9955 and 0.9995, exceeding those of the Freundlich isotherm. Additionally, the adsorption data suggest that the displacement of AMX and DCF ions shows monolayer coverage on the surface of the LS–AC–SG nanocomposite. Therefore, the results adequately fit the Langmuir model.

#### Dubinin–Radusekevisch–Kanager Isotherm

This model is really well-suited for the Gaussian energy distribution and adsorption processes that were carried out on a heterogeneous surface. D–R equation is as follows [49]:

ln q = ln q_(D-R)_ − ßε^2^ (6)

ε = RT ln(1 + 1/C_e_) (7)

where q_(D-R)_ (mg·g^−1^) stands for the theoretical adsorption capacity, ß represents the activity coefficient (mol^2^ kJ^−2^) (the mean sorption energy), ε—the Polanyi potential, R stands for the ideal gas constant (0.008314 kJ mol^−1^ K^−1^), T emulates the absolute temperature in K. E (kJ mol^−1^), denoted as the free energy change, is as follows:

E = 1/(2ß)^1/2^ (8)

The E value can help recognize the reaction’s kind. If E < 8 kJ mol^−1^, it will be expected that the physical forces may affect on the adsorption process. If E is within 8–16 kJ mol^−1^, the sorption takes place by chemical ion exchange. Additionally, the sorption process could be determined by particle diffusion should E be >16 kJ mol^−1^ [50]. The D–R model simulation data are listed in Table 4. E values were 0.7150 and 1.265 kJ mol^−1^ for AMX and DCF ions absorption onto the LS–AC–SG nanocomposite. Therefore, should E be <8 kJ mol^−1^, the sorption is influenced by physical adsorption [51].

### 2.5. Sorption Thermodynamics

To assess the thermodynamic feasibility and spontaneous nature of the process of adsorption, the thermodynamic factors including the standard enthalpy (∆H°), free energy (∆G°) and entropy (∆S°) were assessed to determine the thermodynamic action of the AMX and DCF adsorbed onto the LS–AC–SG nanocomposite. The results were recorded at different temperatures (25, 40, 50 and 60 °C). The following equations were used to determine the thermodynamic factors [51,52]:

∆G° = −RT ln K_d_ (9)

∆G° = ∆H° − T∆S°(10)

ln K_d_ = −∆H°/RT + ∆S°/R (11)

where R is the gas constant (8.314 J mol^−1^ K^−1^), T is the absolute temperature (K) and K_d_ is the distribution coefficient. The Gibbs free energy was computed using Equation (9). Furthermore, ∆G° could be determined from ∆H through Equation (10). Through Equation (11), the thermodynamic variables ∆S° and ∆H° were gauged (from the intercept and slope). The data revealed that the AMX and DCF ions amount uptake by nanocomposites was diminished inversely to the temperature surge. Meanwhile, the surge in temperature enhanced the contaminants’ solubility in a bulk solution to a greater degree compared with that of the adsorbent particles [53]. The thermodynamic sorption effect of AMX and DCF ions on the LS–AC–SG nanocomposite factors is demonstrated in Table 4.

Negative values of ∆G° indicate the feasibility and spontaneity of the adsorption process. Moreover, negative values of ∆H° likewise indicate that the adsorption of AMX and DCF onto the LS–AC–SG nanocomposite was exothermic. Negative values of ∆S° for the LS–AC–SG nanocomposite showed that randomness diminished at the solid–liquid interfaces due to how AMX and DCF adsorbed onto the adsorbents’ surfaces, showing that the adsorption was energetically stable [54]. The sorption was of a physical nature for ΔG° values less than 80 kJ mol^−1^. However, it could have been chemical when ΔG° ranged within 80–400 kJ mol^−1^ [55]. In Table 4, ΔG° values are demonstrated; AMX and DCF sorption was of a physical nature. These outcomes concur with the D–R isotherm.

## 3. Experimental Procedures

### 3.1. Materials

Extrapure activated carbon, limestone purchased from Al-Gomhoria Company (Al-Mansoura, Egypt), sodium alginate and calcium chloride (CaCl_2_) obtained from Sigma-Aldrich were among the materials used. Amoxicillin and diclofenac (500 ppm) were supplied by a Amon company, Cairo, Egypt. The chemical structures of amoxicillin trihydrate and diclofenac are presented in Table 5. Hydrochloric acid (HCl) and sodium hydroxide (NaOH) were used for pH adjustment of the sample. All of the compounds were of commercial quality and had not been purified.

### 3.2. Preparation of LS–AC–SG Nanocomposites

In order to formulate the 2% (*w/v*) adsorbents of sodium alginate, 3 g of activated carbon and 7 g of limestone were mixed in 100 mL of distilled water. The combination was stirred with a mechanical stirrer and heated on a hot plate to 80 °C. When the mixture of activated carbon, limestone and alginate attained a homogeneous condition, it was dripped through a syringe injector into 0.3 M of calcium chloride to form beads. In order to obtain hardened beads, the beads were submerged in a calcium chloride (CaCl_2_) solution for 12 h. Washing the adsorbents with distilled water several times removed the excess unbounded calcium chloride from the adsorbent surface.

### 3.3. Surface Characterization of the Nanocomposites

#### 3.3.1. Instruments

FTIR spectroscopy was used to examine the nanocomposites using potassium bromide (KBr) employing a Genesis-II FTIR spectrometer (ALT, San Diego, CA, USA) at the wavelength of 400–4000 cm^−1^. In addition, scanning electron microscopy (SEM) was performed using an Inspect S (FEI Company, Eindhoven, The Netherlands) armed with an energy-dispersive X-ray analyzer (EDX, Quanta 200, FEL, Eindhoven, the Netherlands). Transmission electron microscopy (TEM) was carried out to measure the particle sizes of the materials employing the JEM-HR-2001 model (JEOL, Akishima, Japan) and accelerating voltage of 200 kV. The mineralogical structure of the powdered materials was determined using X-ray diffraction (XRD) and logged on a Philips PW 1050/70 diffractometer (Philips, Amsterdam, The Netherlands) using a Cu–Kα source with a post-sample Kα filterant, a scanning speed of 1 s/step, a range of 5 to 50 2θ° and a resolution of 0.05°/step. An Agilent HPLC 1200 Infinity instrument armed with a photodiode array detector (Agilent Technologies, Waldbronn, Germany) was employed to reveal the presence of AMX and DCF in aqueous solutions. The chromatograms were recorded at 280 nm. An Agilent Zorbax Eclipse Plus C18 column (3.5 mm, 150 mm, 4.6 mm) (Agilent, Newport, CA, USA) was operated at oven temperature of 25 C. The mobile phase was a mixture of 40% water (mobile phase A) and 60% acetonitrile (mobile phase C). A flow rate of 1.0 mL/min was used. BEL and SORB max (Made in Japan) helped measure the area of the surface and pH tuning was carried out using an OHAUS STARTER 2100 pH meter (Pine Brook, NJ, USA) [58].

#### 3.3.2. Adsorption Studies

The batch sorption experiments were performed by stirring a known amount of the sorbent with an aqueous solution of amoxicillin and diclofenac of the required concentration with a mechanical stirrer in a 100 mL capped flask. Firstly, a particular amount of the sorbent was combined with 25 mL of the sorbate solution and shaken for a sufficient period of time to allow for sorption equilibrium. The mixtures were then filtered through filter paper, and the concentration of the antibiotic and the drug in the solution was measured using HPLC. The effect of many parameters on sorption was examined by varying contact time t (10, 20, 30, 40, 60 min), initial concentration of the pharmaceutical solution in the range of 100–1000 ppm, and the initial pH of the drug solution (2, 5, 6, 10) was used and adjusted with 0.1 M NaOH and HCl. Using the following equation, the proportion of antibiotics and drug sorption was estimated by the flowing equations: (12) and (13) [59].

(12) $$\mathrm{Adsorption}\;\mathrm{capacity}\;q_{e}=\frac{\left(C_{0}-C_{e}\right)V}{W}$$

(13) $$\mathrm{Removal}\;\mathrm{efficiency}\;\%=\frac{\left(C_{0}-C_{e}\right)}{C_{0}}\times 100$$

where q_e_ (mg/g) refers to the equilibrium adsorption capacity, C_0_ and C_e_ denote the initial and equilibrium concentrations (mg/L) of AMX and DCF ions, respectively, and V (L) and W (g) refer to the volume of the solution and the weight of the adsorbent.

## 4. Conclusions

The LS–AC–SG nanocomposite was effectively devised and employed to eliminate AMX and DCF ions from aquatic solutions illustrated through FTIR, SEM, BET adsorption–desorption measurements and TEM. The LS–AC–SG nanocomposite functioned well in the removal of AMX and DCF ions. Moreover, it was quite stable at high temperatures. Sorption capacity largely depends on the solution’s pharmaceutical concentration and pH value. The optimal pH for the adsorption of AMX and DCF ions from aqueous solutions was carefully chosen to be 2. The Langmuir adsorption and pseudo-first-order rate equation were used to fit the adsorption and kinetic models of AMX and DCF ions onto the LS–AC–SG nanocomposite. The thermodynamic factors were calculated, where the reaction was established to be spontaneous and exothermic. The sorption of the LS–AC–SG nanocomposite was physical. The long-term stability of LS–AC–SG in a low pH solution, in particular in a pH 2 solution, was stable over the full time period. The devised composites showed the potential to be implemented as an adsorbent in water treatment applications to displace AMX and DCF ions from contaminated water.

## Figures and Tables

**Table 1:** The data of the surface area, pore volume and average pore radius.

Sample	Surface Area (m^2^ g^−1^)	Pore Volume (cm^3^ g^−1^)	Pore Radius (nm)
LS–AC–SG	27.859	2.469	1.772

**Table 2:** Kinetic modeling with the PFOR, PSOR and Mories–Weber equations.

Kinetic Models	Parameter	Amoxicillin (AMX)	Diclofenac (DFC)
	q_e_, exp (mg·g^−1^)	249	246
PFOR	q_e_, cal (mg·g^−1^)	316.2	398.1
	K_ads_ (min^−1^)	0.00104	0.00075
	R^2^	0.9952	0.9959
PSOR	q_e_, cal (mg·g^−1^)	416	526
	K_2_ (g mg^−1^ min^−1^)	0.00007	0.00003
	R^2^	0.7006	0.6816
Mories–Weber	K_d_ (mg·g^−1^ min^0.5^)	93.6	86.8
	R^2^	0.9838	0.9878

**Table 3:** Sorption isotherms.

Kinetic Isotherm	Parameter	Amoxicillin (AMX)	Diclofenac (DFC)
Langmuir	q_e_, exp (mg·g^−1^)	249	246
	K_L_ (L mg^−1^)	0.8182	0.2284
	R^2^	0.9955	0.9995
Freundlich	K_F_ (mol*^n^*^−1^ L*^n^* g^−1^)	177.7	98.327
	*n*	2.123	3.703
	R^2^	0.9745	0.9265
D–R model	E (kJ mol^−1^)	0.7150	1.265
	q_(D-R)_ (mg·g^−1^)	306.4	316.6
	R^2^	0.9535	0.9955

**Table 4:** Thermal parameters for the adsorption of 500 ppm of AMX and DCF ions using 0.05 g/25 mL of the nanocomposite at pH 2 and a contact time of 40 min.

Parameter	T (K)	A%	ln K_L_	∆H° (kJ·mol^−1^)	∆S° (J·mol^−1^·K^−1^)	∆G° (kJ·mol^−1^)	R^2^
Amoxicillin (AMX)	298	99.6	4.8	−90.01	−257.3	−11.89	0.9904
	313	97	2.7			−7.02	
	323	94	2.3			−6.17	
	333	91	1.6			−4.42	
Diclofenac (DFC)	298	98.4	3.4	−52.78	−145.9	−8.42	0.9952
	313	95	2.2			−5.72	
	323	92	2			−5.37	
	333	90	1.5			−4.15	

**Table 5:** Physicochemical properties of diclofenac and amoxicillin.

	Diclofenac (Sodium Salt) [56]	Amoxicillin (AMX) [57]
Structure	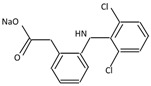	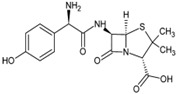
Molecular formula	C_14_H_10_Cl_2_NNaO_2_	C_16_H_19_N_3_O_5_S·3H_2_O
Usage	Analgesic, anti-inflammatory	Antibiotic
Molecular weight (gmol^−1^)	318.13	419.45
pKa	4.3	2.4, 7.4, 9.6
Water solubility (gL^−1^) at 25 °C	2.37	1–3

## Data Availability

Data on the compounds are available from the authors.

## Sample Availability

Samples of the compounds are available from the authors.
